# Assessment of a massive open online course (MOOC) incorporating interactive simulation videos on residents’ knowledge retention regarding mechanical ventilation

**DOI:** 10.1186/s12909-021-03025-8

**Published:** 2021-12-01

**Authors:** Tài Pham, François Beloncle, Lise Piquilloud, Stephan Ehrmann, Damien Roux, Armand Mekontso-Dessap, Guillaume Carteaux

**Affiliations:** 1 Service de Médecine Intensive-Réanimation, AP-HP, Hôpital de Bicêtre, Hôpitaux Universitaires Paris-Saclay, Le Kremlin-Bicêtre, France; 2 Université Paris-Saclay, UVSQ, Univ. Paris-Sud, Inserm U1018, Equipe d’Epidémiologie respiratoire intégrative, CESP, 94807 Villejuif, France; 3 Département de Médecine Intensive - Réanimation, CHU d’Angers, Vent’Lab, Université d’Angers, Angers, France; 4 Adult Intensive Care Unit, University Hospital and University of Lausanne, Lausanne, Switzerland; 5 CHRU de Tours, Médecine Intensive Réanimation, CIC INSERM 1415, CRICS-TriggerSEP FCRIN Research Network, CHRU de Tours and Centre d’Etude des Pathologies Respiratoires (CEPR), INSERM U1100, Université de Tours, Tours, France; 6 AP-HP, Hôpital Louis Mourier, DMU ESPRIT, Médecine Intensive-Réanimation, F-92700 Colombes, France; 7 Université de Paris, UFR de Médecine, F-75018 Paris, France; 8 Assistance Publique-Hôpitaux de Paris, CHU Henri Mondor, Service de Médecine Intensive Réanimation, F-94010 Créteil, France; 9 Université Paris Est-Créteil, Faculté de Santé, Groupe de Recherche Clinique CARMAS, F-94010 Créteil, France; 10 INSERM U955, Institut Mondor de Recherche Biomédicale, F-94010 Créteil, France

**Keywords:** MOOC, Residents, Mechanical ventilation, Respiratory physiology

## Abstract

**Background:**

Understanding respiratory physiology and mechanical ventilation is a challenge for healthcare workers, particularly, medical residents. A team of French-speaking experts developed an innovative MOOC incorporating interactive simulation-based videos and serious games aiming at improving knowledge and skills in mechanical ventilation. Our objective was to evaluate the long-term knowledge retention regarding key concepts presented in this MOOC.

**Methods:**

French residents registered for the MOOC 2020’s winter session were invited to participate in a two-step study. The first step consisted in evaluating students’ pre-course knowledge of respiratory physiology and mechanical ventilation fusing a 20 five-item multiple choice questions test with a total score ranging from 0 to 100. For the second step, the same students answered the same test (after shuffling the questions) six months after the completion of the course. We assessed the impact of this MOOC on the students’ knowledge retention by comparing pre-course and post-course scores.

**Result:**

Of the 102 residents who agreed to participate in the study, 80 completed the course and their mean ± SD pre-course score was 76.0 ± 8.0. Fifty-one respondents also completed the second and their post-course score was significantly higher than the baseline one (83.1 ± 7.3 vs. 77.5 ± 7.6, *p* < 0.001). Scores of the first and second rounds did not differ upon comparing respondents’ background specialty or number of years of residency. For the vast majority of individual questions (96%), the success rate was higher at the post-course than at the pre-course assessment.

**Conclusion:**

An innovative MOOC incorporating simulation-based videos was effective in teaching medical residents basic mechanical ventilation knowledge and skills, especially in the field of respiratory physiology and ventilatory modes. We observed effective long-term knowledge retention with a higher score at the post-course assessment six months after the completion of the course compared with the pre-course score.

**Supplementary Information:**

The online version contains supplementary material available at 10.1186/s12909-021-03025-8.

## Introduction

Most patients admitted to Intensive Care Units (ICUs) receive invasive or non-invasive mechanical ventilation [1, 2]. When used properly, mechanical ventilation improves the outcome of patients with respiratory failure. Conversely, inappropriate ventilator settings can increase mortality [3].. The operational training of healthcare professionals on mechanical ventilation is therefore a crucial issue. However, such training is complex as it comprises acquiring a broad knowledge (of respiratory physiology, ventilatory modes, lung protective ventilation, etc.) and apprehending its related application [4]. As a result, residents generally do not gain the essential knowledge and skills required to properly use mechanical ventilation no matter how long they stay in the ICU and despite theoretical training [5]. A recent review on mechanical ventilation training during graduate medical education showed that: 1) trainees are generally dissatisfied with mechanical ventilation training, and 2) the best results are obtained with integration of simulation [6]. Of more, simulation based training was superior to traditional training even when assessed at the end of ICU rotation with daily exposure to mechanical ventilation in both arms [7]. However, simulation-based training is time consuming and might not be suitable for wide-scale training programs. That is a fact we observed worldwide during COVID-19 pandemics where the influx of critically-ill patients with respiratory failure exceeded daily ICU capacities in terms of beds and specialized human resources [8].

Most students now have open access to knowledge using their connected laptops and smartphones [9, 10]. New forms of teaching and learning methods have become alternatives to the traditional face to face classroom teaching [11]. In early 2007, a report from the American Medical Colleges’ Institute for Improving Medical Education highlighted the interest of multimedia learning [12]. A study on 91 medical students in their third year showed that applying multimedia presentations enhanced learning in medical education [13]. Worldwide universities have developed distance-learning programs with different models including Massive Open Online Course (MOOC). The latter is a web-based courses accessible by anyone, anywhere in the world as long as an internet connection is available. In another word, MOOC is suitable for wide-scale training. In 2011, the first MOOC released by Stanford University on artificial intelligence attracted 160,000 online registrants [14]. The recent COVID-19 pandemic strongly favored distance learning and many universities and teachers developed strategies to provide education despite the limited or impossible access to the classroom [15]. MOOCs and other types of distance programs grew exponentially in the past years especially in 2020 during COVID-19 pandemics [15].

In 2018, a group of French-speaking mechanical ventilation experts created a training course tackling the challenge of broadly transmitting not only fundamental knowledge but also operational skills in mechanical ventilation: MOOC “EIVASION” (***E****nseignement* ***I****nnovant de la* ***V****entilation* ***A****rtificielle par la* ***S****imulat****ION*,** or Innovative simulation-based course in mechanical ventilation). This MOOC specifically uses interactive videos displayed via a high-fidelity simulator of mechanical ventilation, to create a dematerialized simulation environment through which operational skills can be transmitted. The first session of this course held in 2019 was attended by 4700 registrants, of whom 1057 (22%) successfully completed the whole training. There are numerous MOOCs available worldwide in a variety of fields (*e.g.* social, technology, engineering…). Studies have reported the ability of this type of training to improve knowledge [16–19]. However, in the field of health and medicine, despite numerous MOOC produced [20], only a minority has been dedicated to medical student training or continuing medical education. Assessment of such MOOCs in the field of medical training remains scarce [21]. Additionally, incorporating simulation-based videos in an interactive environment to teach both knowledge and skills has never been assessed. Our goal was to assess the long-term benefit of MOOC EIVASION on terms of residents’ knowledge of basic as well as essential concepts of mechanical ventilation and respiratory physiology.

## Materials and methods

### MOOC EIVASION

MOOC EIVASION is a two-level course taught by 20 experts in artificial ventilation over two five-week sessions: “Artificial Ventilation: the Basics” (https://www.fun-mooc.fr/courses/course-v1:UPEC+169001+session03/about) and “Artificial Ventilation: Advanced Level” (https://www.fun-mooc.fr/courses/course-v1:UPEC+169002+session02/about). This MOOC is available in a self-paced format, i.e. it remains open all year round, which allows learners to customize their learning pace. The original MOOC EIVASION curriculum includes two types of educational videos:

- “classical” e-learning videos (e-Fig. 1): these videos were shot in a studio. A teacher speaks in front of the camera, and tackles theoretical notions from slides or animations embedded in the background.

- “simulation-based” videos (e-Fig. 2): these videos were made using a sophisticated high-fidelity simulator capable of virtually simulating any type of patient on artificial ventilation (RespiSim® Manikin, IngMar Medical, Pittsburg, PA, USA connected to a breathing simulator Active Servo Lung 5000 [ASL5000®]). The patient simulator was connected to a respiratory support device (mechanical ventilator). The teacher stands “at the simulator bedside” to teach the practical application of mechanical ventilation (*e.g*., ventilator settings adjustments) and to interpret the flow and pressure waveforms displayed on the ventilator’s screen). These simulation-based videos were shot with one of the commercially available interactive multi-camera recording system (Omnilive®, Current Productions, Paris, France), allowing the learner to navigate between four simultaneous views with a single click (*e.g.*, teacher, ventilator’s screen, ventilation interface). The aim was to reproduce the expert teaching at the bedside, but available to as many people as possible.

Additionally, some serious games (*i.e.* games designed with a training and teaching purpose) combining several simulation-based videos were regularly provided throughout the course. Typically, a serious game starts with a video simulating a pathological situation where the learner is asked first to tick the right diagnosis of the three proposed answers, and secondly to tock the appropriate therapeutic choice of the three proposed choices. Clicking on any of these choices leads to a new simulation-based video, which shows the learner the effects of their decision, whether correct or not. The learner can retrieve and pick another choice in case the first was wrong. Thus, a single serious game is a combination of four (if limited to the diagnostic step) to seven (if covers both diagnostic and therapeutic steps) simulation-based videos. The first level of MOOC (“Artificial Ventilation: the Basics”) comprises 40 videos (of which 15 simulation-based ones), 43 tests (quizzes), and six serious games containing 26 simulation-based videos.

The present study focuses on assessing the impact of the first level of the MOOC on residents’ long-term knowledge retention.

### Study design

When registering for the first level of MOOC EIVASION (“Artificial Ventilation: the Basics”) 2020’s winter session, participants were asked to answer an anonymous survey composed of 10 questions on their medical and academic background. At the end of the survey, the French intensive care residents were asked to participate in the study to evaluate the benefit of MOOC to their knowledge and skills in respiratory physiology and basic principles of mechanical ventilation.

For the evaluation, the researchers used a test composed of 20 multiple-choice questions. Two members of the MOOC education team (TP, GC) created the questions in consistency with the course program to evaluate key concepts addressed in the three predefined teaching domains: basic physiology (nine questions), ventilatory mode principles (five questions), and ventilator waveforms interpretation (six questions). Each of the 20 questions was designed in a single-best option format, and the learner was asked to choose the best response from five answers or to pick either true or false for other questions. The evaluation was conducted at two different time points: 1) pre-course assessment: just before starting the first MOOC session (January 2020); 2) long-term post-course assessment: six months after the completion of the course (September 2020). For the two sessions, questions were similar but arranged in a different random order. Based on the answers, we computed a score that ranges from 0 (all answers are wrong) to 100 (all are correct).

### Participants

MOOC was opened to anyone, but our goal was to specifically assess its potential benefits to ICU residents. Therefore, of all the participants who answered the initial survey, we only asked residents who were working in ICU or who had spent at least a six-month internship in ICU during their residency to participate in the study.

Participants’ information and the use of their personal data were managed in accordance with the General Data Protection Regulation (GDRP) rules.

### Study endpoints

The primary endpoint analysis compared between the score obtained at the pre-course assessments with that obtained at the long-term post-course assessment. The secondary endpoints analysis included the comparisons between the scores obtained at the pre-course and long-term post-course assessments in three predefined domains, namely basic physiology, ventilatory mode principles, and ventilator waveforms interpretation. Additional endpoints were the effect of participants’ characteristics on pre- and post-course scores, comparison of participants who completed versus those who did not complete MOOC, comparison of pre-course scores between residents who participated in both assessments and those who participated only in the first one.

As the first COVID-19 wave occurred in France between the two evaluations, we added several questions to the long-term assessment in order to assess the following dedicated secondary endpoints: 1) the potential effect of the outbreak on completing MOOC and participating in the study, 2) the potential effect of resident’s involvement in COVID-19 patient’ care on the second assessment, and 3) learner’s perception of the usefulness of MOOC EIVASION in the management of COVID-19 patients, particularly those requiring artificial ventilation.

### Statistical analysis

Continuous variables are reported as mean ± standard deviation (SD) or median [first, third quartiles] and categorical variables as number and percentages. Continuous variables were compared using the Student t test or Wilcoxon rank sum test or paired Wilcoxon rank sum test for paired data. Categorial variables were compared using chi-square or Fisher exact test as appropriate (or McNemar test for paired data of residents who participated in both rounds).

No statistical power calculation was conducted prior to the study and convenience sample was based on available data. No assumptions were made for missing data. Statistical analyses were done with R (version 4.0.1). All *p* values were two-sided, and values less than 0.05 were deemed statistically significant.

## Results

### Participants’ characteristics

**Fig. 1:**
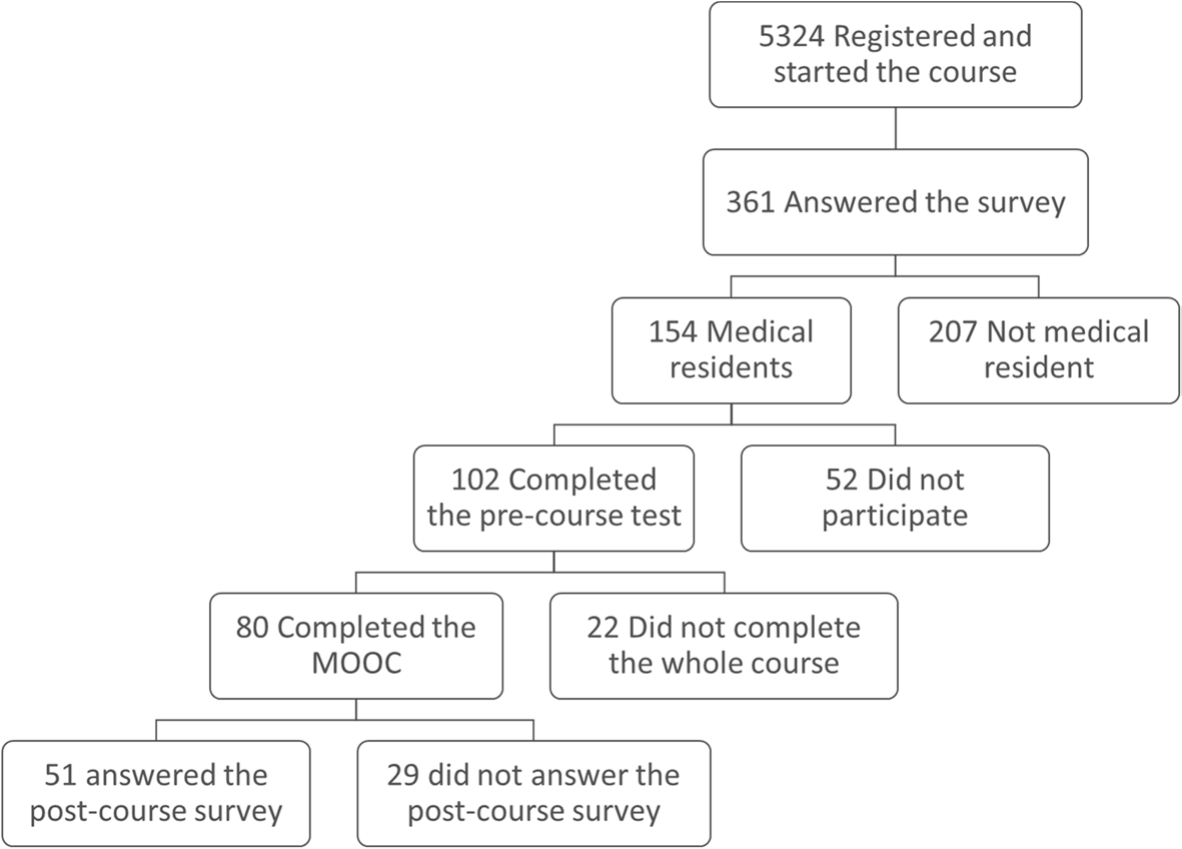
Flowchart of the MOOC participants

Among the 5324 registrants of the 2020’s winter session who started the course, 2668 (50%) completed the whole program. A total of 361 learners answered the survey, including 154 residents, of whom 102 (66%) agreed to participate in the study (Fig. 1).

**Fig. 2:**
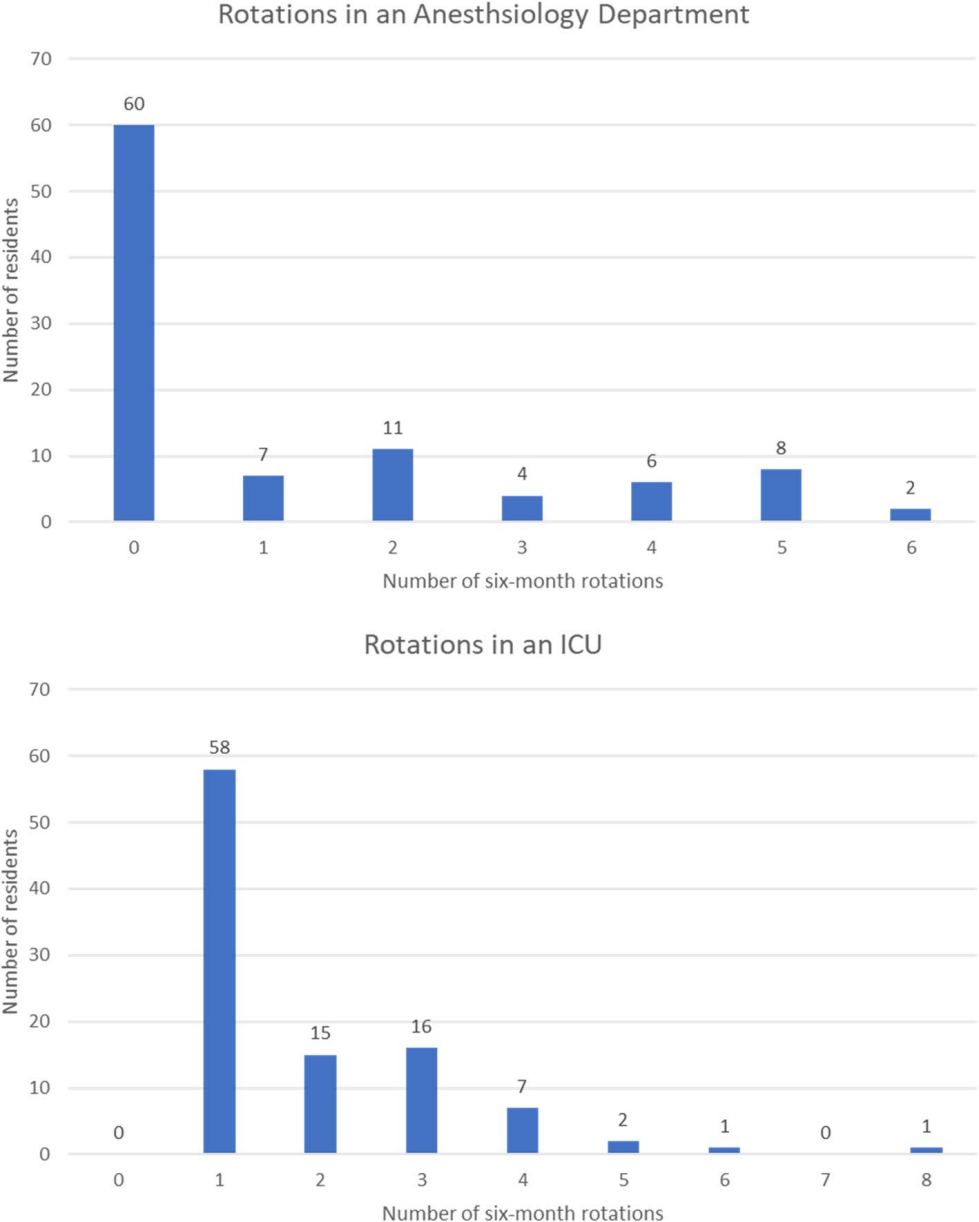
The residency stay in the ICU and anesthesiology department of the pre-course assessment respondents

All respondents had spent at least one semester of their rotations in the ICU, and 38 at least one semester in the anesthesia department (four missing answers). The number of six-month rotations in anesthesiology and ICU is shown in Fig. 2, hence their experience in those fields.

Of the 102 residents who answered the first part of the study (pre-course test), 80 (78%) actually completed the course and 51 (50%) also answered the second assessment performed six months after the end of the course.

Residents who completed the course had spent less time in the ICU than those who did not (*p* = 0.005, Table 1). No other difference was observed between the residents who completed the course and those who did not.

**Table 1:** The demographic characteristics of the residents who participated in the first assessment and completed the course and those who did not complete the course

	All (N = 102)	Did not complete the course (N = 22)	Completed the course (N = 80)	*P*-value
More than two years of residency	56 (55%)	14 (64%)	42 (53%)	0.49
Specialty program				0.37
Intensive Care	11 (11%)	2 (10%)	9 (11%)	
Anesthesiology	48 (49%)	10 (50%)	38 (48%)	
Emergency Medicine	6 (6%)	0 (0%)	6 (8%)	
Other Adult Medical specialties	28 (28%)	5 (25%)	23 (29%)	
Pediatrics	6 (6%)	3 (15%)	3 (4%)	
Current semester (residency)	5 [1;7]	5 [3.25;7]	5 [1;7]	0.23
Nb of six-month rotation in ICU	1 [1;3]	2 [1;3]	1 [1;2]	0.005
Nb of six-month rotation in Anesthesiology Department	0 [0;2]	1 [0;4]	0 [0;2]	0.12

As for the 80 learners who completed the course, the characteristics of the 51 who answered both parts of the study did not differ from those of the 29 who only answered the first test (Table 2).

**Table 2:** The demographic characteristics of the residents who completed the course and participated in the two parts of the study and those who only answered the first assessment

	All (N = 80)	Participated only in the first assessment (N = 29)	Participated in both assessments (N = 51)	*P*-value
More than two years of residency	42 (53%)	19 (66%)	23 (45%)	0.13
Specialty program				0.18
Intensive Care	9 (11%)	3 (11%)	6 (12%)	
Anesthesiology	38 (48%)	9 (32%)	29 (57%)	
Emergency Medicine	6 (6%)	3 (11%)	3 (6%)	
Other Adult Medical specialties	6 (8%)	3 (11%)	3 (6%)	
Pediatrics	3 (4%)	1 (4%)	2 (4%)	
Current semester (residency)	5 [1;7]	5 [3;7]	3 [1;7]	0.42
Nb of six-month rotation in ICU	1 [1;2]	1 [1;2]	1 [1;2]	0.92
Nb of six-month rotation in Anesthesiology Department	0 [0;2]	0 [0;1]	0 [0;2]	0.37

### First round of assessment (before starting MOOC)

**Fig. 3:**
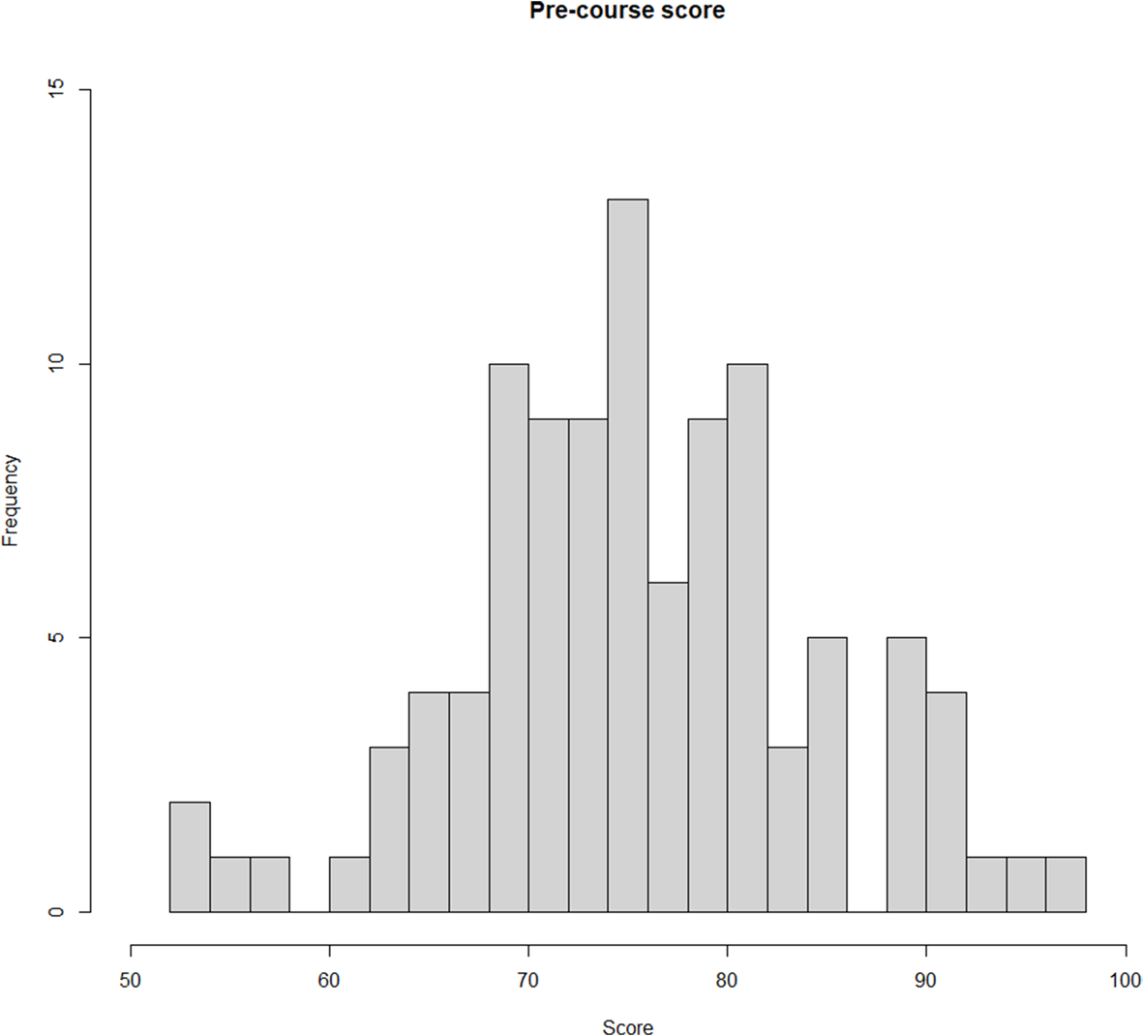
Score of the 102 participants who answered the pre-course test

A total of 102 residents answered the pre-course assessment, with a mean ± SD score of 76.0 ± 8.0 (Fig. 3).

The score was not different between the 46 residents who were in their first two years of residency and the 56 more experienced residents (76.8 ± 8.1 vs. 75.3 ± 9.2, *p* = 0.38). The score was not different between the 58 residents who had spent only one six-month rotation in ICU and the 42 who had spent two six-month rotations or more in ICU (75.0 ± 7.5 vs. 77.5 ± 10.5, *p* = 0.20). Alike, the score was not different between the 48 residents doing their specialty in anesthesiology as compared with the 51 residents of other specialty programs (77.2 ± 8.6 vs. 75.8 ± 8.2, *p* = 0.41).

The questions that had the lowest rates of right answers were those on respiratory motion eq. (17% of correct answers), time constant (31% of correct answers) and relationship between respiratory system elastance and compliance (26% of correct answers).

### Second round of assessment (6 months after completing MOOC)

Eighty residents completed the whole course and answered the first test. The percentage of residents who had spent more than one semester in ICU did not differ between those who answered only the first test and those who answered both (34 and 33%, respectively, *p* = 0.94). The same results applies whether the residents were still in their first two years of residency (34 and 45% respectively, *p* = 0.13) or specializing in anesthesiology (31% vs 57%, *p* = 0.06).

**Fig. 4:**
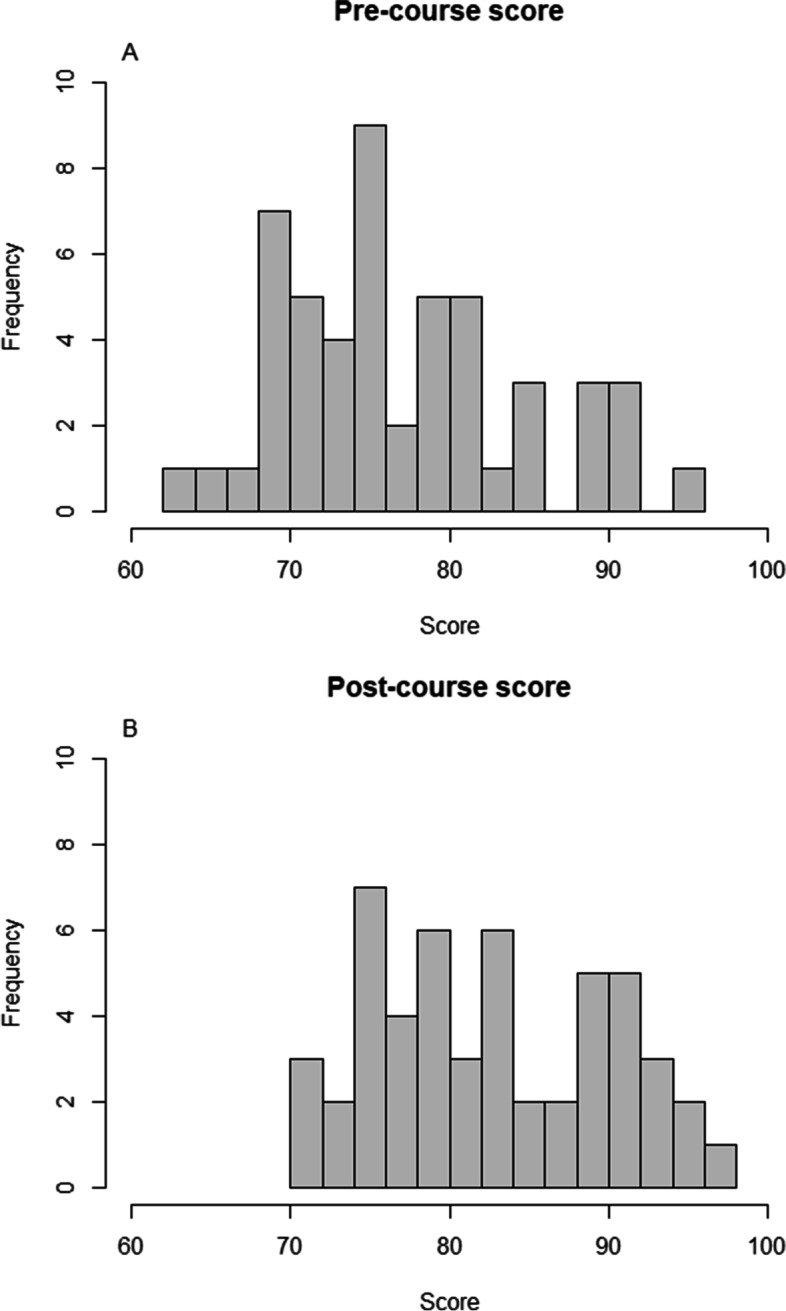
Pre-course score (panel A) and post-course score (panel B) of the 51 residents who answered both

Fifty-one residents answered the post-course test for a score of 83.1 ± 7.3 (Fig. 4). The score did not differ between the 34 residents still in the first two years of their residency and the 17 more experienced (82.9 ± 7.2 vs 83.5 ± 7.7, *p* = 0.78).

The score did not differ whether residents had spent only one semester in ICU or two semesters and more (84.1 ± 6.7 vs. 81.9 ± 7.9, *p* = 0.29). Similarly, no significant difference was found between residents specializing in anesthesiology and those of other specialty programs (84.3 ± 6.7 vs. 81.5 ± 7.9, *p* = 0.19).

The questions with the lowest rates of right answers were those on respiratory motion eq. (33%), the relationship between positive end expiratory pressure (PEEP) and peak inspiratory pressure (45%) and relationship between respiratory system elastance and compliance (49%).

### Comparison between the two rounds

#### Primary outcome

**Fig. 5:**
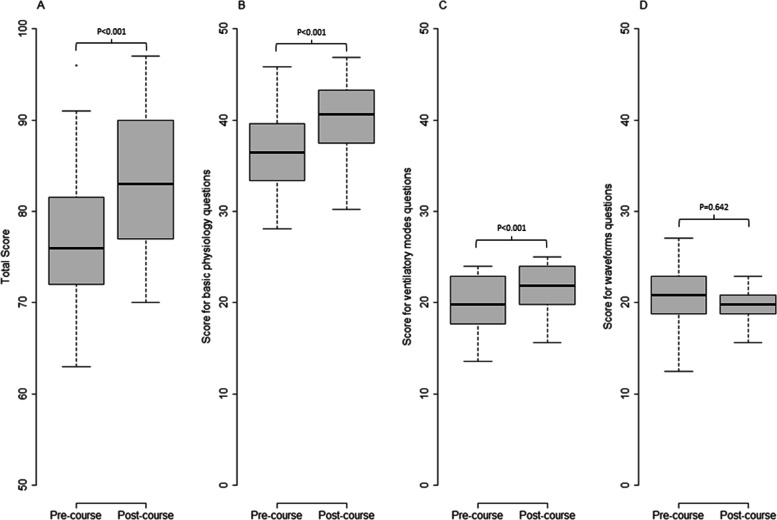
Boxplot comparing pre-course and post-course scores of the 51 residents who completed both rounds. Panel A: total score; Panel B: Score of questions on basic physiology; Panel C: score of ventilatory modes questions; Panel D: score of waveform analysis questions

Concerning the 51 residents who completed both rounds, the score of the second test was significantly higher than that of the first (83.1 ± 7.3 vs. 77.5 ± 7.6, *p* < 0.001, Fig. 5). Their median [IQR] individual scores improvement was 4 [0;8.5] points.

Scores of the second test were significantly higher than those of the first one for questions related to basic physiology (40.3 ± 3.9 vs. 37.2 ± 4.2, *p* < 0.001) and ventilatory modes fundamentals (21.7 ± 2.4 vs. 19.5 ± 2.9, *p* < 0.001) but were not different for questions related to ventilator waveforms interpretation (21.8 ± 2.6 vs. 20.8 ± 3.4, *p* = 0.64) (Fig. 5).

Concerning the three questions for which the rate of correct answers in the first test was the lowest, the residents scored significantly higher “good answers” in the second round for questions on time constant (29 to 61%, *p* = 0.003) and compliance and elastance (29 to 49%, *p* = 0.02). However, it was not statistically different for the question on respiratory motion eq. (17 to 33% *p* = 0.06)).

For each individual question, the rate of correct answer was higher in the second round (reaching statistical significance in 19 questions) except for three questions. Inversely, the rate was significantly lower only for a question regarding the relationship between peak pressure and plateau pressure during pressure assist-control ventilation (decreased from 88 to 68%, *p* = 0.009).

### Potential impact of COVID-19 pandemic

Between the two rounds, 49 (96%) of the participants in the second round had already worked in ICUs to treat COVID-19 patients with respiratory failure. To cope with the surge of patients, 10 (20%) of the respondents declared having more responsibilities than residents usually have (“senior physician-like responsibilities”); 17 (33%) declared having worked in ICU that dealt with more than 50 patients intubated for COVID-19 related respiratory failure, and 14 (27%) declared having personally treated more than 25 of such patients. Most of the respondents (84%, N = 41) reported that they used skills they learned from MOOC EIVASION several times a week. Additionally, 46 (94%) declared that knowledge they got from MOOC EIVASION made them more comfortable upon managing COVID-19 patients, especially the mechanical ventilation settings (98%, N = 48). Scores were not different between the 17 respondents who had worked in ICUs that dealt with more than 50 COVID-19 intubated patients were treated and the 30 who worked in ICUs that dealt with less than 50 (85.1 ± 7.2 vs. 82.1 ± 7.1, *p* = 0.14). Scores were also not different between respondents who had managed more versus less than 25 COVID-19 patients (81.6 ± 5.6 vs. 83.5 ± 7.7, *p* = 0.52). Finally, scores did not differ between respondents who had “senior physician-like responsibilities” during the pandemics or not (83.6 ± 6.5 vs. 83.2 ± 7.4, *p* = 0.86).

## Discussion

### Results summary

Our study showed that teaching the basics of respiratory physiology and mechanical ventilation through an innovative MOOC incorporating dematerialized simulation improved residents’ long-term knowledge retention with higher scores 6 months after course completion. We also showed that this type of teaching is feasible since 50% of the participants completed the entire course. Most respondents found this course helpful in their daily work with ICU-intubated patients.

### Context

To our knowledge, our study is the first to assess the benefit of a MOOC-based training with simulation-based videos for French speaking residents in terms of improving their knowledge and skills in mechanical ventilation. The benefit of simulation-based training in mechanical ventilation has already been reported [7, 22]. Simulation however is expensive, time-consuming, and not suitable for wide-scale training. A study evaluating the effect of a 3-day mechanical ventilation bootcamp showed an increase in learners’ knowledge; but the study had a small sample size (17 students). This type of teaching is also resource demanding: specialized educators were present for three days and the learners’ knowledge was evaluated immediately after the bootcamp, which does not ensure knowledge retention for a longer period [23]. Unlike face-to-face simulation, online education has the potential of reaching a massive number of learners. A recent study conducted on American residents rotating in Pediatric ICU showed that an online virtual mechanical ventilation simulator increased resident’s knowledge on this topic [24]. With short rotation periods, i.e. typically shorter than one month, the residency curriculum in the USA differs a lot from the French residency program and its 6-month rotation schedules. Short-term rotations might benefit more from additional on-line learning because residents do not have the same exposure to clinical practice and time to receive pragmatic education during their rotations. Over the past decade, numerous MOOCs were produced in the field of health and medicine [20]. However, only a minority was dedicated to medical student training or continuing medical education. The development of this type of dematerialized education has been enormously progressed during COVID-19 pandemic. Nonetheless assessment of MOOCs in the field of medical training remains scarce [21]. Our concept of MOOC incorporating simulation-based videos in an interactive environment is highly innovative and oriented to teach both knowledge and skills in mechanical ventilation. We have found no previous report of MOOC incorporating dematerialized simulation into a comprehensive curriculum.

### Performance

Our test was developed to assess residents’ basic ICU-required knowledge of respiratory physiology and mechanical. Participants’ pre-course knowledge was good with a mean score of 76.0 ± 8.0. As this course is not mandatory, residents who registered for the MOOC might have a specific interest in the topic which could explain this relatively high baseline performance. It is not surprising questions on basic physiology had lower scores since that knowledge is more difficult to retrieve at the bedside. Performance at the second round was high and only two questions had a success rate lower than 49%. The first one was reformulating the respiratory motion equation using elastance as a surrogate of 1/compliance. Most respondents selected the correct formula using compliance but did not realize that compliance could be replaced by the inverse of elastance.

The second question that had a low success rate was evaluating the effect of PEEP on peak pressure in volume-controlled mode (the higher the PEEP, the higher the peak pressure). Though the veracity of this question cannot be challenged, this concept is not of paramount importance for most clinical situations at the bedside.

Upon restricting assessment to the residents who answered both assessments, scores of the second round performed six months after the end of the course, were higher and the rate of “correct answer” to each individual question was higher for almost all of them. More precisely, the second test scores were higher for the questions on basic physiology and ventilatory modes principles, but not for questions regarding ventilator waveforms interpretation, a skill more likely dependent on experience. This might indicate that MOOC provided good quality teaching and clarified most of the key concepts required to use mechanical ventilation at the bedside with good retention over a six-month period. The only question that had a significant poorer rate of good answer concerned a ventilatory mode that is rarely used in France.

### Pragmatic usefulness

Unexpectedly, COVID-19 pandemics occurred between the two assessments. The health systems worldwide faced a massive influx of patients with COVID-19 related severe acute respiratory distress syndrome (ARDS). The French ICUs were heavily impacted and almost overwhelmed by the high number of patients. New ICU beds were created, sometimes in unusual locations such as operating rooms or post-surgical recovery rooms. The total number of ICU beds in France almost tripled from 5, 000 to 14, 000 in a very short time [25]. Most of the residents who answered the second round had already participated to COVID-19 patients’ management. They declared that the knowledge and skills obtained via MOOC helped them optimize care of these patients. In many ICUs, the number of clinical shifts as well as the number of night duties had to be increased for all physicians including residents. This might explain why 36% of the residents who started the MOOC did not complete the whole course.

### Limitations

This study has several limitations. First, the tests were created by the MOOC educators, which could have overemphasized the importance of concepts they clearly explained in MOOC and expected the learners to know. Second, the better performance at the second round could be biased since residents were asked the same questions in the pre-course round, a reason for them to focus on these specific concepts upon visualizing them at MOOC. We tried to limit this bias by randomizing the order of questions in the second round and by waiting for a six-month period before the second assessment to test the long-term retention. The uncontrollable event that could have affected learner’s performance at the second assessment was COVID-19 pandemic. All respondents had treated such patients and almost one third declared having personally managed more than 25 patients on mechanical ventilation due to COVID-19. This could have improved their skills and knowledge of respiratory physiology and mechanical ventilation. On the other hand, the use of mechanical ventilation is expected to be part of their daily practice in their ICU or anesthesia rotation anyway. It has been shown that simulation-based training performed before ICU rotation was superior to traditional training for bedside skills in mechanical ventilation which was assessed after the ICU rotation, i.e. after continuous exposure to mechanical ventilation in both arms [7]. Additionally, Singer *et al.* reported that simulation-trained first-year residents outperformed traditionally trained third-year residents upon assessing bedside skills in mechanical ventilation [22]. This is consistent with our findings that scores were not influenced by the number of previous rotations in ICU. Overall, it seems that dedicated training in mechanical ventilation may affect knowledge and skills regardless of clinical experience gained during residency. Eventually, the magnitude of score improvement observed in our study is consistent with that observed with simulation-based training [7]. However, whether MOOC EIVASION-based training may give different results from simulation-based training remains unknown. Finally, one can suppose that residents who answered both assessments had a higher interest and more experience on this topic. Though we did not measure enthusiasm to the subject, we did not find any difference in the demographic characteristics of those who answered only the first survey as compared with those who answered both.

## Conclusion

An innovative MOOC incorporating simulation-based videos was effective in teaching medical residents basic mechanical ventilation knowledge and skills, especially in the field of respiratory physiology and ventilatory modes. It increased long-term knowledge retention with higher scores six months after the completion of the course as compared with the pre-course results. This distance learning not only improved theoretical knowledge but may have also improved pragmatic skills as most respondents found this course helpful to treat ICU-intubated patients. Pre- and post-course scores did not appear to be influenced by learners’ clinical experience in either year of residency, number of ICU rotations, or number of patients managed during COVID-19 pandemic. This highlights the importance of teaching the knowledge and skills required to provide appropriate care to patients who need mechanical ventilation, independent of resident’s academic education during their ICU rotations. Future work should compare MOOC EIVASION-based training with simulation-based training in terms of the acquired knowledge and bedside skills in mechanical ventilation.

## Supplementary Information

**Additional file 1.**

## Data Availability

De-identified data can be requested to the study scientific committee.

## Abbreviations

ARDS: acute respiratory distress syndrome
COVID-19: Coronavirus disease 2019
EIVASION: Enseignement Innovant de la Ventilation Artificielle par la SimulatION
ICU: Intensive Care Unit
MOOC: Massive Open Online Course
PEEP: positive End-Expiratory Pressure
SD: Standard Deviation
