# Correction to: Assessment of a massive open online course (MOOC) incorporating interactive simulation videos on residents’ knowledge retention regarding mechanical ventilation

**DOI:** 10.1186/s12909-021-03089-6

**Published:** 2022-01-25

**Authors:** Tai Pham, Francois Beloncle, Lise Piquilloud, Stephan Ehrmann, Damien Roux, Armand Mekontso-Dessap, Guillaume Carteaux

**Affiliations:** 1 Service de Médecine Intensive-Réanimation, AP-HP, Hôpital de Bicêtre, DMU 4 CORREVE Maladies du Cœur et des Vaisseaux, FHU Sepsis, Groupe de Recherche Clinique CARMAS, Le Kremlin-Bicêtre, France; 2 Université Paris-Saclay, UVSQ, Univ. Paris-Sud, Inserm U1018, Equipe d’Epidémiologie respiratoire intégrative, CESP, 94807 Villejuif, France; 3 Département de Médecine Intensive - Réanimation et Médecine Hyperbare, CHU d’Angers, Vent’Lab, Université d’Angers, Angers, France; 4 Adult Intensive Care Unit, University Hospital and University of Lausanne, Lausanne, Switzerland; 5 Médecine Intensive Réanimation, Inserm CIC 1415, Réseau CRICS-TRIGGERSEP F-CRIN network, CHRU de Tours and INSERM, Centre d’Etude des Pathologies Respiratoires (CEPR) UMR 1100, Université de Tours, Tours, France; 6 AP-HP, Hôpital Louis Mourier, DMU ESPRIT, Médecine Intensive-Réanimation, F-92700 Colombes, France; 7 Université de Paris, UMR1137 IAME, INSERM, F-75018 Paris, France; 8 Assistance Publique-Hôpitaux de Paris, CHU Henri Mondor, Service de Médecine Intensive Réanimation, F-94010 Créteil, France; 9 Université Paris Est-Créteil, Faculté de Santé, Groupe de Recherche Clinique CARMAS, F-94010 Créteil, France; 10 INSERM U955, Institut Mondor de Recherche Biomédicale, F-94010 Créteil, France

**Correction to: BMC Medical Education (2021) 21:595**

**https://doi.org/10.1186/s12909-021-03025-8**

Following publication of the original article [1], we have been informed that the authors Damien Roux, Armand Mekontso-Dessap and Guillaume Carteaux were incorrectly affiliated.

Also, the author Armand Mekontso-Dessap was incorrectly tag. We have tagged his name as follows:

First Name: Armand

Last Name: Mekontso-Dessap

The author group has been updated above and the original article [1] has been corrected.
