# Supplementary material for: Assessment of a massive open online course (MOOC) incorporating interactive simulation videos on residents’ knowledge retention regarding mechanical ventilation
Source: BMC Med Educ. 2021 Dec 1;21:595. doi: 10.1186/s12909-021-03025-8 (PMC8635324; doi:10.1186/s12909-021-03025-8)
Supplement: Supplementary file 1 — Additional file 1. [file 12909_2021_3025_MOESM1_ESM.docx]

**ELECTRONIC SUPPLEMENTARY MATERIAL**

**Assessment of an innovative Massive Open Online Course (MOOC) integrating interactive simulation videos on resident’s knowledge regarding mechanical ventilation**

Tài Pham, François Beloncle, Lise Piquilloud, Stephan Ehrmann, Damien Roux, Armand Mekontso Dessap, Guillaume Carteaux

e-Figure 1


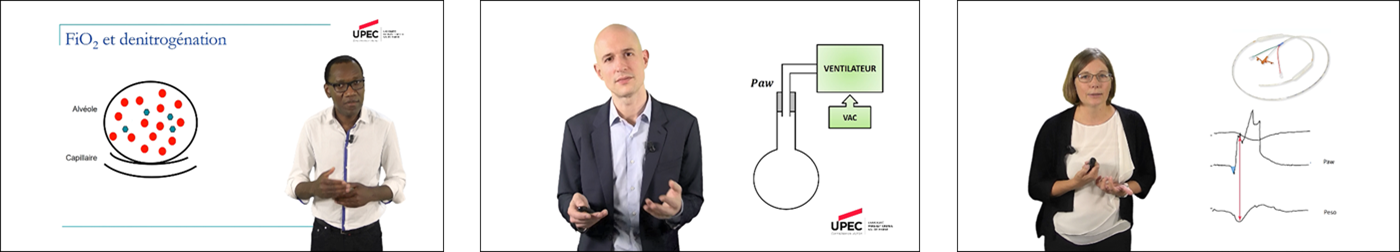

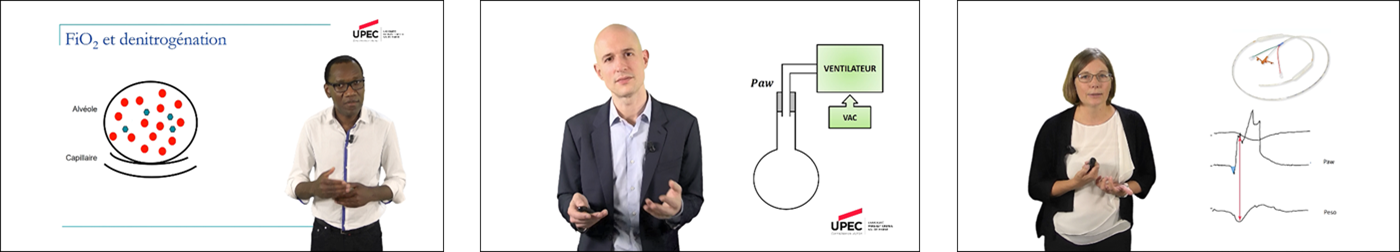


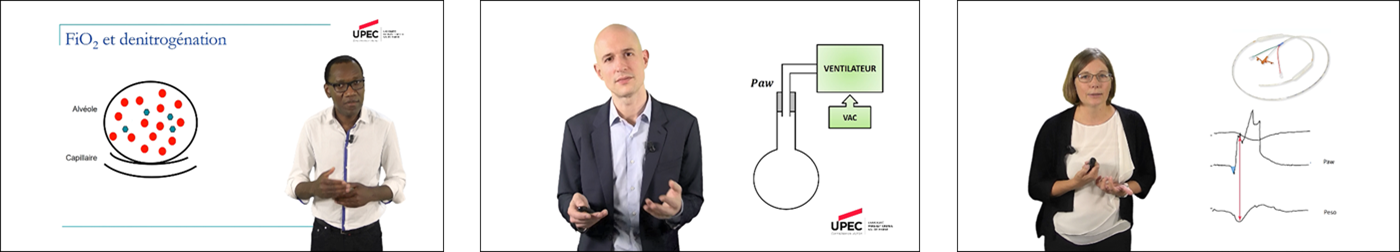


Representative screenshots of “classical” e-learning videos. These videos were shot in a studio; the teacher spoke in front of the camera, and taught theoretical notions from slides or animations embedded in the background.

e-Figure 2


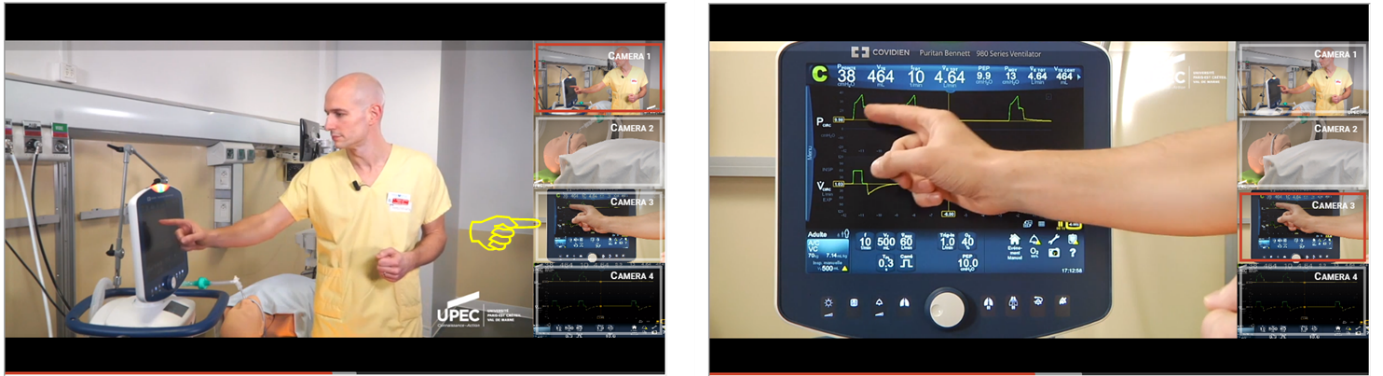


Representative screenshots of a “simulation-based” videos. These videos were made using a sophisticated high-fidelity simulator capable of virtually simulating any type of patient under artificial ventilation. The simulator, mimicking a true patient, was connected to the respiratory support (invasive mechanical ventilation in this video). The teacher was positioned “at the simulator bedside” to teach the practical application of mechanical ventilation (*e.g*., ventilator settings adjustments, interpretation of flow and pressure waveforms available on the ventilator’s screen). These simulation-based videos were shot with an interactive multi-camera recording system allowing the learner to navigate between four simultaneous views – available on the right side of the screen – with a single click (*e.g.*, teacher, ventilator’s screen, ventilation interface). In this figure, both screenshots represent the same video. The screenshot on the right is the view from the ventilation interface after clicking on the corresponding shot (the click is represented by the yellow hand on the left screenshot).
